# The Perils of Autobiography: Evidence, Meaning, Reliability, and Context in the Historiographical Canon on Carl Ransom Rogers, 1950s–2020s

**DOI:** 10.1007/s12124-026-09982-3

**Published:** 2026-03-09

**Authors:** Catriel Fierro

**Affiliations:** History of Science Institute (iHC), Autonomous University of Barcelona, Barcelona, Spain

**Keywords:** Psychobiography, Historiography of science, Philosophical history of psychology, Epistemology of historical research, Client-centered therapy

## Abstract

This article examines the historical canon on Carl Ransom Rogers (1902–1987) as a case study in the epistemological and methodological shortcomings of ‘insider’ psycho-biographical narratives in the historiography of psychology. Most accounts of Rogers, written by Rogerian practitioners, rely heavily on a limited cluster of biographies and autobiographical recollections treated as transparent self-reports confirming the personal, developmental origins of his theories. I suggest such dependence has produced an ‘echo-chamber’ historiography: a closed system of citation, repetition, and uncritical reverence for inherited narratives privileging Rogers’ first-person accounts. Through methodological criticism of published historical scholarship and archival reconstruction of Rogers’ mid-century writings and professional context, I show that his autobiographical texts emerged from specific institutional, rhetorical, and personal circumstances rather than spontaneous self-disclosure. By situating these materials within broader debates on the historiography of the human sciences, I argue that the Rogerian canon exemplifies how disciplinary self-legitimation can distort historical explanation by collapsing history, memory, and celebration. I conclude by calling for a reflexive historiography grounded in archival evidence, methodological pluralism, and epistemic caution.

## Philosophical Sensibility and Narrative Nuance in Biographical Historiography

“[A]s a clinician I feel that the individual reveals himself in the present and that a true history of his psychogenesis is impossible.” (Rogers, 1967, p. 343).

As a scholarly inquiry into the past through primary sources and documentary evidence, the field of History of Psychology is deeply entangled with philosophical and epistemological concerns, both in its procedures and subject matter (Burman et al., 2025). These concerns include the structure of historical knowledge, the nature and reliability of evidence, the semantics of historical narratives, the nature and dynamics of science, and the place of epistemic values in historical explanation (Stanford, 1986). Given these philosophical underpinnings, the interpretive work of the historian of psychology extends beyond the collection of facts: it requires reflection on how those facts become known, what warrants their use as evidence, and what meanings can be reasonably extracted from them (Teo, 2013). To improve their research practices and the quality of their narratives, historians must combine detailed, contextually grounded analysis, often based on archival work, with second-order reflection on the assumptions guiding historical inference (Araujo, 2017). Without this reflexive dimension, the historiography of psychology risks reproducing the same epistemological habits that shaped psychological theories and practice, collapsing the distinction between historical and psychological analysis.

Among its many subfields, biographical historiography, and especially narratives adopting psychological or developmentalist explanations, has proved particularly vulnerable to such methodological and philosophical tensions (Elms, 1997; Mülberger, 2025). As a style seeking to link an individual’s life with the development of his or her psychological ideas or practices, psycho-biographical historiography has often been scrutinized and severely criticized by historians of science (Söderqvist, 2007; Sokal, 2010). Yet the genre remains widespread among psychologist-historians, especially practitioners such as personality psychologists and psychotherapists.

Writing from within psychology’s professional community, practitioner-historians have often approached historical sources not as problematic artifacts but as self-evident truths, and historical subjects not as contingent actors but as moral or intellectual exemplars. Assuming that personality theories are shaped by “a complex interplay between subjective and personal factors” (Atwood & Tomkins, 1976, p. 176), psychologist-historians often examine the lives of historical figures to *explain* the cognitive content of their theories or techniques (Anderson, 2005; Fancher, 1998). This developmentalist logic, which Alan Elms aptly termed “compulsive originolog[y]” (1997, p. 73), rests on the assumption that “relationships exist between a thinker’s professional work and his or her personal life” (Demorest, 2014, pp. x–xi), and that these are clear, evident and unproblematic. Consequently, such accounts, often couched in the language of early experiences, introjection, empathy, conflict, and identification, often risk turning historical reconstruction into disciplinary commemoration (Wettersten, 1975).

Given the plurality of purposes served by historical narratives, it would be unproductive to advance a rigid, a priori normative distinction between ‘good’ and ‘bad’ historical writing and analysis, for instance by dismissing ‘originological’ approaches as simply deficient forms of history. Part of the scientific community still adheres to the view that, as has been recently suggested, “[c]elebratory historians play a crucial role in strengthening their communities, irrespective of their failures to interrogate the [historical] material.”^1^ Indeed, the existence of a disciplinary ‘canon’ in a specific field, defined by historian of science Simon Schaffer (1996, p. 207) as “the corpus of exemplary texts that provide a standard of that discipline”, is intrinsic and structural to the organization and dynamics of the sciences. Furthermore, as Collyer (2010) has noted for the social and medical sciences, the process of canon construction stabilizes otherwise fluid identities, secures disciplinary legitimation, and promotes professionalization: in brief, it invents and fixes a tradition.

Still, Historiography itself —as a discipline with identifiable standards and knowledge claims— and, mutatis mutandis, the diverse Histories of Science that constitute its specialized or partial historiographies, is partly normative by definition, which necessarily makes the analysis, scrutiny, and comparison of historical narratives a legitimate part of its field of inquiry (Stanford, 1986). As García (2019) has recently put it, the existence of celebratory and canonical accounts of the history of a specific science, and their adoption by practitioners of psychology as normative frameworks, while necessary and unavoidable, does not negate that in terms of factuality, accuracy, or truthfulness, those accounts are usually “a poor conception of the past and the present of the discipline” (p. 175). This, tied the fact that distinctive historiographical formations do exist, each one shaped by specific professional aims, epistemic commitments, and intended audiences, does not preclude criticism, but actually calls for informed, thorough, and minute scrutiny.

This productive and rather ‘generative’ approach to celebration has been readily reflected in the way historians have reacted to the aforementioned ‘insider’ historiographic custom. Historians have attempted to build bridges between theory and biography, repeatedly emphasizing the methodological differences between biography and hagiography (e.g. Ball, 2012; Sokal, 2010) and showing how new archival sources can correct or outright replace canonical narratives (Araujo, 2014; García, 2015). More to the point, thorough studies of clinical and personality psychologists such as Henry Goddard (Zenderland, 2003), Carl Gustav Jung (Shamdasani, 2005), Hans Eysenck (Buchanan, 2010) and Aaron Beck (Rosner, 2014), among many others, demonstrate that such authors, their lives and their ideas must be treated as evidentiary problems rather than objects of uncritical commemoration (for an overview, see Marks, 2017). From this perspective, professional trajectories emerge at the intersection of institutional settings, intellectual networks, and rhetorical self-presentations, none of which can be straightforwardly inferred from autobiographical testimony alone.

This tension between biographical-developmentalist and historical-contextualist approaches structures the historiography of noted personality theorist and psychotherapy researcher Carl Ransom Rogers (1902–1987). Indeed, Rogers has been consistently described as both a central figure and a defining force in modern psychotherapy ever since the 1930s (Crisp, 2025). Nonetheless, despite his prominence and a handful of recent, critical analyses in historical scholarly journals (e.g., Fierro, 2021; Guenther, 2022), the Rogers canon, understood here as the standard narrative of his life and works assumed by default by Rogerian practitioners and non-specialist historians alike, remains largely biographical in style, hagiographic in aim, internalist in audience, and developmentalist in focus (Marks, 2017; Rosner, 2018). Much of this literature has been produced by practitioner-authors sympathetic to the humanistic or client-centered tradition, who have used biography as a mode of professional legitimation or intellectual appreciation. These accounts commonly trace the origins of Rogers’ ideas to his Protestant, Midwestern upbringing and rely heavily on his own autobiographical statements and retrospective reflections, often treating them as transparent records rather than as rhetorically and institutionally situated documents. Epitomizing this tradition, Jack Martin’s recent work on Rogers has argued that because psychology “concerns human action and experience,” “the life experiences, beliefs, and values of psychologists cannot easily be detached from their psychological thinking and practices” (Martin, 2025, p. 262). Beverly Palmer (2025, p. 530), while disagreeing with Martin’s specific conclusions about Rogers’ personal development, endorses the same premise by seeking “theme[s] in Carl Rogers’ life” as explanatory of his cognitive claims.

Such conviction, expressed early on in Rogers’ collaborator-turned-biographer Julius Seeman’s claim that “a theory in the human sciences is autobiography” (1990, p. 381), has become constitutive of Rogers scholarship: the assumption that childhood experience, maturation of the self, and personal development provide the primary explanation for theoretical origination and systematic development. The present article hence examines the difficulties that arise when autobiography is treated as historical evidence rather than as a historical phenomenon requiring explanation, as well as the epistemological assumptions underlying the frequent identification of history, theory, and memory. Taking as a starting point the assumption, articulated by García, that the critical study of a historical canon “complements the history of knowledge production, as it shifts the focus from the authors’ context to the process of reception [and the way in which] they were read, discussed, and used” (2015, p. 9. Translation mine), I offer a critical reconstruction of the Rogerian biographical canon as a historiographical genre, identifying its characteristic sources, narrative strategies, and recurrent interpretive moves. Second, I demonstrate how archival materials, especially unpublished correspondence and manuscript drafts, allow Rogers’ autobiographical statements to be re-situated within the shifting professional, institutional, and personal contexts in which they were produced. Drawing on discussions of historical methodology (Burnham, 2003; Sokal, 2010), contextual historical research (Forner et al., 2025; Zenderland, 2003), and the epistemology of historical research in psychology (Burman, 2022; Teo, 2013), the article shows how a small corpus of Rogers’ autobiographical writings, reproduced by a limited cluster of biographies and amplified by a wide array of practitioner-authored texts, has come to dominate interpretations of his life and work.

By foregrounding the historical situatedness of autobiographical materials, this article seeks to open a dialogue between practitioner-centered biographical traditions and contextualist historical approaches while underlining the importance of archival work for the Rogerian tradition. Rogers’ case is instructive not because it is unique, but because his unusually extensive autobiographical production, combined with the relative paucity of sustained archival research on his career, makes highly visible the epistemic stakes involved in treating autobiography as historical evidence. By tracing how developmentalist biography became the dominant Rogerian genre, and by offering counterexamples grounded in archival reconstruction, the article ultimately outlines what a more critically reflexive and philosophically informed historiography of psychology might entail.

## The Historiographical Rogerian Canon: Limited Sources and Fossilized Narratives

Over the past fifty years, and dozens of obituaries notwithstanding, biographical and historical writings on Carl Rogers have appeared regularly, roughly one per year, across clinical, counseling, and psychotherapy outlets, with the pace accelerating in the last decade to at least a total fifteen new publications.^2^ These works are typically authored by psychologist-historians, often Rogerian practitioners or personality theorists, and published in humanistic or client-centered outlets and venues, including presses tied to person-centered, non-academic or religious communities: as Nye (1981, p. 143) noted early on, Rogers’ strongest following has lied among those “not trained professionally at the doctorate level in psychology.”

This professional milieu has decisively shaped the historiography, as the detachment of Rogerian thought from mainstream academia has distanced its chroniclers from university-based research centers and fostered a literature marked by shared aims, interpretive assumptions, and a remarkably uniform documentary base (see Lietaer, 1990; Raskin, 1990). These psycho-biographical narratives of Rogers have seemingly served several important functions: legitimating a therapeutic tradition, buttressing core meta-theoretical ideas on science, evidence and research, fostering collective identity, and articulating continuity between personal character, clinical attitude, and theoretical innovation. Yet these same functions have placed constraints on the kinds of historical questions such narratives can plausibly address, particularly when autobiographical materials are read as transparent, straightforward windows into theoretical origins rather than as historically situated texts.

Although Rogers historiography is extensive and varied, nearly all published accounts gravitate toward his childhood and formative years. While few titles are as explicit as Dolliver’s (1995a) *Carl Rogers’s Personality Theory and Psychotherapy as a Reflection of His Life Experience and Personality*, most analyses follow a similar path, tracing Rogers’ theoretical ideas to his early experiences in the affluent Chicago suburb of Oak Park during the early 1900 s and mid-1910s.^3^ These reconstructions aim to provide explanatory contexts expressed as formative ideas, attitudes, beliefs, or dispositions for Rogers’ later concepts of personality development, behavior change, therapist acceptance, and unconditional positive regard (Crisp, 2025; Dalton, 2022; Demorest, 2014; Joseph, 2025). In the most recent example of this trend, Martin (2025, p. 249) has claimed that Rogers’ “thwarted self-expression in childhood,” shaped by strong parental control, a very religious household, and in-family teasing lay at the core of his later theoretical commitments. Martin concludes that Rogers’ “struggles with congruence in his therapeutic relationships and professional and personal life are directly related to his theories of personal development and core psychotherapeutic conditions” (2025, p. 263).

The field’s typical prevailing developmentalist custom can be traced to the late 1970 s, when Rogerian therapists and personality theorists, while conceding that psychological practice “frequently occurs without adequate awareness of the origins of clinical approaches”, began assuming that this missing “adequate awareness” could be achieved by focusing on Rogers’ early life and formative years (Sollod, 1978, pp. 93–95). Voicing many other Rogerian counselors, Sollod pointed out that analyzing these experiences was “relevant to understanding the roots of [Rogers’] personality” and thus explanatory of his later theoretical system (Sollod, 1978, p. 95). Shortly thereafter, Rogerians began perceiving Rogers as “rooted in his particular cultural background” and backtracking client-centered therapy to his “personal experiences” (Nye, 1981, p. 115). This search for personal clues quickly became widespread during the 1980 s, as therapists increasingly looked to Rogers’ early life for the origins of his professional approach (Cain, 1987; Fuller, 1982). By the 1990s, the assumption that, in Seeman’s terms, “a theory in the human sciences is autobiography” (Seeman, 1990, p. 381) had become firmly entrenched in Rogers scholarship.

Far from remaining a mere heuristic suggestion, Rogerian psychologist-historians transformed this idea into a methodological axiom, assuming that “[t]heorists’ life experiences and personalities often influence their theories” (Dolliver, 1995a, p. 113). As Robert Fuller observed in his analysis of Rogers’ religious background, the implied belief here was and has been that “psychological thought” (Rogers’ included) “is a derivative of some larger Weltanschaung [sic] or symbolic universe” (1982, p. 25). By translating this *psychological* idea into a historiographic claim, Rogerians often posit that Rogers’ religious upbringing “imparted interests that were to factor significantly into his life-long opposition to scientific positivism” and that his parents’ narrow beliefs “simultaneously disposed him to understand personal integrity as something that arises through resistance to external authority” (Fuller, 1982, p. 25).

It is beyond the scope of this reconstruction to determine whether causal explanations or even meaningful historical relationships can be established between such tenuously documented episodes, scattered across wide temporal spans and interpreted largely by ‘insider’ psychologist-historians who admit that “we can [only] *speculate*” about Rogers’ motives and cravings (Dolliver, 1995a, p. 120, emphasis mine). Nor is it my aim to test the logical or evidentiary foundations of developmentalist biographical historiography *as a whole*, especially considering that even biographically-inclined historians of science have adopted claims that are ‘softer’, tentative, and more correlational than causal (e.g., Fancher, 1998; Sokal, 2010; see also Shamdasani, 2005 and Mülberger, 2025). My aim instead is to recognize this long-standing developmentalist trend in Rogers scholarship, scrutinize its methods and documentary base, and expose its limitations to foster more historically grounded and plausible narratives.

Close reading of historical scholarship suggests the Rogers canon lacks sustained engagement with primary sources, archival collections, or proper historical documentation. Rather, the standard narratives rely on official or (more rarely) unauthorized biographies of Rogers, and his own published recollections, oral histories, and autobiographical memoirs. A critical analysis of these two documentary pillars, and of the contextual frameworks in which both biographies and autobiographies were produced, is offered in the following two sections.

## House of Cards and Echo Chambers: Secondary Sources as Narrative Cornerstones

A salient characteristic of Rogers scholarship is its heavy dependence on a very specific cluster of biographies. The first edition of Howard Kirschenbaum’s *On Becoming Carl Rogers* (1979) was the standard reference work throughout the 1980 s and 1990 s, especially in English-language literature, as analyzed below. A second, short English biography, Brian Thorne’s *Carl Rogers* (1992), appeared soon after, followed by British psychologist David Cohen’s *Carl Rogers: A Critical Biography* (1997). Cohen’s was the first biographical account written by a non-Rogerian and self-described ‘critical’ outsider, and it in turn prompted Kirschenbaum to rework his own book, published ten years later as *The Life and Work of Carl Rogers* (2007). Finally, an oral history titled *The Quiet Revolutionary*, marketed as “a virtual autobiography of Carl Rogers” (Rogers & Russell, 2002, p. xxiv) and analyzed in the next sections, was published in 2003.

Four points about these biographies are worth emphasizing, since they function as the cornerstones of the developmentalist view of Rogers’ historiography. First, they have been readily accepted as standard, accurate portrayals of Rogers’ life, personality and work *by default*— this is, almost automatically and without systematic scrutiny. As personality psychologist Amy Demorest admitted in her psycho-biographical study of Rogers, “it is from Kirschenbaum’s biography and David Russell’s oral history that we can extract a picture of Rogers’ life” (Demorest, 2014, p. 146). Yet the conditions of production of these sources are rarely examined, if acknowledged: Kirschenbaum became very close to Rogers and his wife while preparing his book, and Russell’s oral history was recorded under highly specific institutional and interpersonal circumstances, as analyzed below. The interpretive decisions shaping Cohen’s (1997) narrative, often sardonic and polemical, and the lack of in-text references to sources, have also remained largely unexplored, even though Kirschenbaum references Cohen repeatedly in the second edition of his biography (2007).

Still, as Dolliver aptly observed, these works, especially Kirschenbaum and Cohen’s, are repeatedly treated as “provid[ing] revealing information.”^4^ That these two narratives are often mutually exclusive in their statements and conclusions has gone mostly unnoticed. Indeed, these two works neatly represent the ‘authorized’ and the ‘independent,’ the hagiographic and the sensationalist extremes that have defined nearly half a century of debate about Rogers and his ideas.

Such automatic trust in biographical sources springs partly from the Rogerian movement’s respect for oral tradition (Rogers & Haigh, 1983). Biographers and psychologist-historians commonly assume that resorting to recollections by Rogers’ acquaintances and collaborators “in order to better understand other facets of [Rogers’] life” is a legitimate and dependable approach (Heppner et al., 1984, p. 14). Just as Rogers’ direct disciples relied on personal memories to interpret his work, later writers rely on a narrow group of biographies, especially Kirschenbaum’s, as default repositories for statements about Rogers’ “‘tenacious,’ ‘dogged,’ [and] ‘forceful’” demeanor (Crisp, 2025, p. 1), the “beginning” of client-centered therapy (Cain, 1990), the seed of Rogers’ “development [through] an inexorable movement” (Seeman, 1990, p. 382), or the “one central idea” toward which the author (not Rogers) feels “[a]ll of Rogers’ convictions point” (Cooper, 1989, p. 19). Biographies are invoked either to channel Rogers’ authentic voice or, less often, to humanize him. In both cases, they are treated as the primary access point to the origin of his theory: an origin that, within the developmentalist framework, invariably lies in childhood and personal growth.

Somewhat paradoxically, these biographies are not only limited in number but also remarkably thin in *contextual* analysis. As historians of psychology have long emphasized, historical narratives acquire meaning only within wider constellations of sources and contexts, for only archives can “provide anchors to stabilize” the changing extension and intension of historical texts (Burman et al., 2025, p. 3), and only context can provide meaning for an historical document *qua* part of a broader whole (Teo, 2013, p. 845; see also Zenderland, 2003). That biography is not incompatible with sophisticated contextual analysis has also been repeatedly pointed out (e.g. Mülberger, 2025). However, *On Becoming Carl Rogers* (1979) offers merely a few paragraphs on Rogers’ family life in the early 1900s (mostly recollections of a seventy-year-old Rogers) and provides no sustained social or cultural analysis. Furthermore, the absence of footnotes or source references makes critical verification impossible.

On the other hand, Cohen’s *Carl Rogers: A Critical Biography* (1997), though written partly as a reaction against Kirschenbaum’s hagiography, rarely specifies its own sources for the colorful descriptions of family conflicts and customs. Only in *The Life and Work of Carl Rogers* (2007) does Kirschenbaum begin to cite his sources and provide limited context for early-twentieth-century Midwestern life. Even there, however, his reconstruction of early 1900s Oak Park –the cornerstone of developmentalist interpretations– leans heavily on historical studies of another native son, Ernest Hemingway (e.g. Grimes, 1996). Indeed, Kirschenbaum (2007), Cohen (1997, pp. 25-27), and more recently Martin (2025, p. 249) and Dalton (2022) have all claimed that Rogers and Hemingway (allegedly) shared the same suburban environment. Yet Rogers scholarship has uncritically extrapolated from Hemingway’s Oak Park experience, assuming it to be comparable to Rogers’ own—a decision which, as I have argued elsewhere (Fierro, 2023) is hardly tenable. This borrowing has produced a ready-made sociocultural backdrop at the expense of case-specific research.

Third, the *extent* to which the same few biographies have come to dominate both primary and secondary literature must also be underlined. In Martin’s (2025) recent article on Rogers’ lifelong pursuit of authenticity, roughly 60% of his seventy-three citations refer to two sources: thirty-five to Kirschenbaum and eight to Cohen. Similarly, of the ninety references in Dalton’s (2022) analysis of transcendental influences on Rogers, almost a quarter are to Kirschenbaum alone. Cain’s (1987) and Demorest’s (2014) studies of Rogers’ early life are similar in their repeated reliance on the same claims found in Kirschenbaum’s biographies, which they leave unexamined. Of course, this dynamic is not unique to Rogers scholarship, nor is it necessarily the product of methodological negligence. Indeed, repetition and stabilization of historical narratives can inform pedagogy, foster identity formation, and encourage disciplinary legitimation. Still, this intense citation clustering demonstrates how developmentalist explanations depend on texts that, paradoxically, provide little contextual or archival depth– the very elements such explanations require. In other words, internal coherence is achieved only through a comparatively low degree of evidentiary expansion.

Core to my analysis, this recursive pattern creates a muffled, layered, self-referential and often recursive narrative: interpretations nested within quotations nested within further interpretations, until Rogers himself becomes difficult to locate, if present at all. For instance, Martin (2025, p. 249) quotes Cohen (1997, pp. 24–25), who appears to quote Rogers describing himself as “something of a book worm,” “extremely absent-minded,” and “inept in social situations.” Yet a brief examination reveals of Cohen’s biography reveals the author is offering his own interpretation rather than Rogers’ words. Likewise, when Dolliver (1995a, p. 116) cites Rogers “at age 13” saying “I never rebelled in behavior very much, not until much later,” close inspection reveals he is actually quoting Kirschenbaum (1979, p. 15), whose biography contains no endnote identifying the statement’s source (but which the reader might assume comes from the then-70-year-old Rogers). Similarly, when Dolliver (1995a, p. 119) quotes Rogers recalling that in his boyhood home “emotional displays were inappropriate,” the phrasing again derives from Kirschenbaum (1979, p. 31), who offers no quotation marks or clarifying reference.

Thus, the problem here is not simply that secondary sources are overused, but that their use is rarely interrogated. And since direct engagement with archival manuscripts is rare, such ambiguities go unresolved in several other cases (e.g. Cain, 1987, pp. 494-495). This creates a historiographical “house of cards” which rests not only on the fragility of individual claims, but on the cumulative weight placed on a narrow and self-reinforcing body of literature. Psychologist-historians who claim to “turn to Rogers’ personal life” to find “sources for his theoretical concepts” (Demorest, 2014, pp. 145-146) end up chasing a mirage crafted by earlier biographers. In other words, when Rogerians turn to biography as a substitute for history, they are not uncovering evidence of how “Rogers’ own personal experiences may have made him more sensitive to some phenomena” (Demorest, 2014, p. 145) but reproducing inherited narratives.

Finally, the biographies’ reception has entrenched these problems through uncritical praise. Kirschenbaum’s work has been described as a “magnificently detailed and empathic biography” (Dalton, 2022, p. 239), as “a comprehensively extensive, balanced, and meticulously researched biography of Carl Rogers [which] must rank as one of the best biographies of any psychologist” (Martin, 2025, p. 257), and as “the definitive biography of Rogers” (Joseph, 2025, p. 208). Cohen’s biography, though often characterized as a “more judgmental interpretation” (Martin, 2025, p. 252), is nonetheless taken as reliable, as shown in his being repeatedly referenced throughout the canon. Regardless of the biographies’ merits, this cumulative validation produces a closure effect: when biographical accounts are treated as definitive, authors begin to perceive historical revision with suspicion, even feeling “that the world did not need another book about Carl Rogers” (Joseph, 2025, p. 207).

Taken together, the tendencies of this pattern –automatic acceptance, minimal contextualization, recursive citation, and celebratory reception– have produced a historiographical canon rich in reverence but poor in renewal. The aim of highlighting this pattern is not to dismiss the contributions of earlier authors, nor to deny the legitimate (although debatable) functions of practitioner-authored historiography. Rather, it is to clarify the *conditions* under which certain narratives about Rogers have acquired their authority in order to explore new avenues and retrace those already surveyed. Having made visible the echo-chamber dynamics of secondary citation, we can now ask not only what has been said about Rogers’ life and work, but how those statements have been stabilized, transmitted, and naturalized over time. This, in turn, opens a much-needed space for reconsidering Rogers’ own autobiographical statements as the evidentiary foundations on which his canon has been built.

## A Disarming Candidness: Rogers’ Autobiographical Recollections as Historical Sources

If secondary sources function in the Rogerian canon as narrative cornerstones, then autobiographical writings constitute its foundational stratum. Indeed, much of what circulates in biographical accounts of Rogers ultimately derives from a small set of first-person texts that have been treated as unusually transparent windows into his inner life, personality, and intellectual development. And it is precisely the extent to which these statements have been taken at face value (secondary only perhaps to the ‘Freudian legend’ or myth) which makes the Rogerian canon an interesting and fruitful historiographical case study.

Rogerians have repeatedly expressed their interest in knowing Rogers “in quite some personal ways,” especially since it is assumed that his personality, attitudes, and tendencies would lead to a “greater comprehension of the creative processes employed in the formulation of theories of personality and psychotherapy.”^5^ Consequently, when attempting to explain the roots of Rogers’ theoretical formulations, biographers and practitioner-historians have focused in particular on his “strict ascetic and religious family,” his alleged “considerable sublimation into work at an early age,” his capacity for “sustained effort of an intellectual scientific nature,” and his “early isolation” (Sollod, 1978, pp. 94–96). However, they have done so almost exclusively within the limits of what Rogers himself disclosed in his own autobiographical statements, as professional historians of psychotherapy have recently pointed out in a critical tone (e.g. Marks, 2017, p. 12).

The four autobiographical sources most often cited in works about Rogers are (1) his personal recollections and musings published as the opening chapter of his national bestseller *On Becoming a Person* (Rogers, 1961), (2) his first formal autobiography, written as a book section for a collection of autobiographies of psychologists in 1967 (Rogers, 1967), (3) his reflections on the origins of his philosophy of interpersonal relations, published in *A Way of Being* (Rogers, 1980), and (4) the oral history conducted by David Russell between 1985 and 1987 and published fifteen years later (Rogers & Russell, 2002). Although some authors combine several of these sources to reconstruct Rogers’ early years, personality, demeanor, and beliefs (e.g., Crisp, 2025; DeCarvalho, 1991; Demorest, 2014; Dolliver, 1995a; Moore, 2025; Palmer, 2025), most rely on only one: either the 1961 chapter (e.g., Atwood & Tomkins, 1976; Bastien, 1968/2018; Dalton, 2022; Dolliver, 1995b; Mruk, 2023; O’Hara, 1995; Seeman, 1990; Sollod, 1978; Thorne, 1992; and Joseph, 2025) or the 1967 autobiography (Cain, 1987; Fuller, 1982).^6^ These texts are commonly used to emphasize Rogers’ family, home environment, and social milieu in the wealthy Chicago suburb of Oak Park as theoretical explanatory factors for his ideas.

At first glance, these autobiographical writings seem to justify such reliance. They display Rogers’ well-known stylistic perks: sharp argumentative style, lucid prose, elegant directness, and seemingly disarming honesty. When describing his upbringing, Rogers established what would become the mold for most later historiography about his life. In 1961 he recalled:

I was brought up in a home marked by close family ties, a very strict and uncompromising religious and ethical atmosphere, and what amounted to a worship of the virtue of hard work. […] They were also, in many subtle and affectionate ways, very controlling of our behavior. It was assumed by them and accepted by me that we were different from other people – no alcoholic beverages, no dancing, cards or theater, very little social life, and much work. (Rogers, 1961, p. 5)

In his more formal but still personal 1967 autobiography, Rogers painted a similar portrait of his childhood:

My parents had both been reared on farms and were highly practical, “down to earth” individuals. […] [T]hey both tended to be rather anti-intellectual, with some of the contempt of the practical person toward the long-haired egghead. They both worked very hard and, more important than this, had a strong belief in the virtue of work. There was almost nothing that a little hard work would not cure. My mother was a person with strong religious convictions, whose views became increasingly fundamentalist as she matured. […] My father was involved too in the family prayers, church attendance and the like, but in a less emotional way. They were both devoted and loving parents, giving a great deal of time and energy to creating a family life which would “hold” the children in the way in which they should go. (Rogers, 1967, p. 344)

Rogers offered yet another, more theory-ladden version of these memories in his later writings. Published toward the end of his life, the 1980 chapter of *A Way of Being*, adapted from a 1972 lecture to the Association for Humanistic Psychology, sought to trace how his belief system had evolved “until it is now almost the antithesis of what I was taught –and believed– in my youth” (Rogers, 1980, p. 27). Here, Rogers was more transparent in employing his own theoretical vocabular to make sense of his personal development:

In a narrowly fundamentalist religious home, I *introjected* the *value attitudes* toward others that were held by my parents. Whether I truly believed in these I cannot be sure. I know that *I acted on* these values. I think the attitudes toward persons outside our large family can be summed up schematically in this way: “Other persons behave in dubious ways which we do not approve in our family. Many of them play cards, go to movies, smoke, dance, drink, and engage in other activities, some unmentionable. So the best thing to do is to be tolerant of them, since they may not know better, but to keep away from any close communication with them and to live your life within the family.” […] My thoughts, my fantasies, and the few *feelings I was aware of* I kept to myself. (Rogers, 1980, pp. 27–28. Emphasis mine)

This seemingly disarming candidness has led psychologist-historians to assume immediacy and transparency in Rogers’ accounts, claiming that “Rogers clearly was exceptional in promoting others’ ability to understand his personal side.”^7^ Fuller’s remark that “fortunately” Rogers “has been unusually candid about the biographical and cultural contexts of his professional work” (1982, p. 25) and Dolliver’s belief that he “made many efforts to aid others’ ability to understand him in quite personal ways” (1995a, p. 113) are paradigmatic of this interpretive stance: one in which respect and esteem frequently blur into admiration and reverence. These assessments are emblematic of a broader stance in which admiration for Rogers’ ethos of transparency, openness and honesty shades into confidence in the integrity of his recollections and the evidentiary status of his published accounts.

As a result, these and many similar first-person statements have often been lifted and taken at face value, treated as “primary sources” granting direct access to “the inner world” of the subject (Demorest, 2014, p. xi). It is therefore not surprising that psychologist-historians have felt justified in asserting that Rogers’ parents’ “ethic of hard work” remained with him throughout life (Dolliver, 1995a, p. 115; Lakin, 2009, p. 245), that he drew scientific insights and experimental culture from agricultural labor (Cain, 1987, p. 477; O’Hara, 1995, pp. 41–42; Joseph, 2025, p. 19), that he somaticized the “stress” generated by emotional repression (Atwood & Tomkins, 1976, p. 171; DeCarvalho, 1991, p. 21; Dolliver, 1995a, p. 116), that he developed a critical stance toward authority early on (Dolliver, 1995a, pp. 116; 1995b), and that his break from parental control revealed “the negative psychological consequences of interiorizing others’ conditions of worth,” leading to his characteristic “psychological theorizing” (Dolliver, 1995a, p. 120; see also Atwood & Tomkins, 1976, pp. 171–172). From a historical (and historiographical) standpoint, the issue here is not whether these interpretations are implausible, but rather how they have been justified and grounded. A sizeable share of these accounts (e.g., Cain, 1987; Seeman, 1990; Sollod, 1978) do little more than assemble Rogers’ own statements, organize them under preselected themes, and draw a connecting line dictated by an a priori biographical hypothesis. Moreover, all assume these recollections as factual and project them backward in time, concluding, as O’Hara (1995) does, that “[l]ike most great scientists, researchers, or students of nature,” Rogers “was *already* practicing his calling as a young boy,” studying “the mysteries of the universe” through moths and cattle in his Chicago suburb (p. 41. Emphasis mine).

The affective and professional proximity between Rogers and many of his biographers complicates this picture even further. Authors have been straightforward about their admiration for Rogers’ “folksy, semi-autobiographical style” (Martin, 2025, p. 247) and “warm, personal, and often intimate” manner (Heppner et al., 1984, p. 14). Many biographers have openly acknowledged Rogers as a “centrally important figure” in their own lives and credited Rogers’ “empathic responses” as the reason they became Rogerians (Dolliver, 1995a, p. 112). Scholars who interviewed Rogers in person described him as having “an eye-opening quality that poignantly illuminates the human condition” (Heppner et al., 1984, p. 14), and recalled that in meeting him as a historical subject they “above all got to experience [his] honesty” (Rogers & Russell, 2002, p. xxv). They also admitted approaching Rogers as “an old friend who has aided us in our growth,” while also feeling “the anxieties associated with meeting a childhood hero or an idolized mentor” (Heppner et al., 1984, p. 14), and confessed developing a “close relationship” with the subject during their biographical work (Kirschenbaum, 1979, p. xvi). In brief, psychologist-historians often feel struck by Rogers’ demeanor and reflected in his history and journey: self-help author, business coach, and therapist Stephen Joseph voices many of his colleagues when describing his recent book on Rogers’ humanistic approach (commissioned by Oxford University Press) as “a work about my own discovery of Rogers’ work” (Joseph, 2025, p. 10).

Such commitments are neither surprising nor illegitimate, but they do underscore the importance of examining autobiographies (and secondary sources such as historical reconstructions) as historically situated literary products. In the field of History of Science, candidness, however compelling, is not synonymous with transparency, reliability or completeness. Again, a frank recognition of this does not diminish the value of autobiographical primary sources, but it does invite a more analytical, distant, and even suspicious approach. As the following sections argue, engaging with these materials as artifactual documents shaped by memory, context and disciplinary interests opens new interpretive vistas which remain mostly inaccessible within a strictly developmentalist and ‘originological’ frame.

### Autobiography, Artifactuality and Incompleteness

The Rogerian canon has largely treated autobiographical statements as “disclosive self-reports.”^8^ Yet historiographical debates, especially in the history of the human and clinical sciences, have shown that such intentionalism is interpretively misleading and often factually untenable (Shamdasani, 2005; Rosner, 2018).

Autobiographies are often the products of deliberation, editing, and rhetorical shaping– processes involving not only the authors but also editors, publishers, and relatives (Elms, 1997). Assuming interpretive distance and viewed critically, Rogers’ autobiographical writings display the familiar limitations of first-person reports and must be read as *artifactual* rather than transparent documents. Indeed, their apparent immediacy is revealed as illusory, and the relationship between statement and referent is shown as mediated, negotiated, and contingent, especially when contrasted with archival sources and unpublished material.

**Fig. 1:**
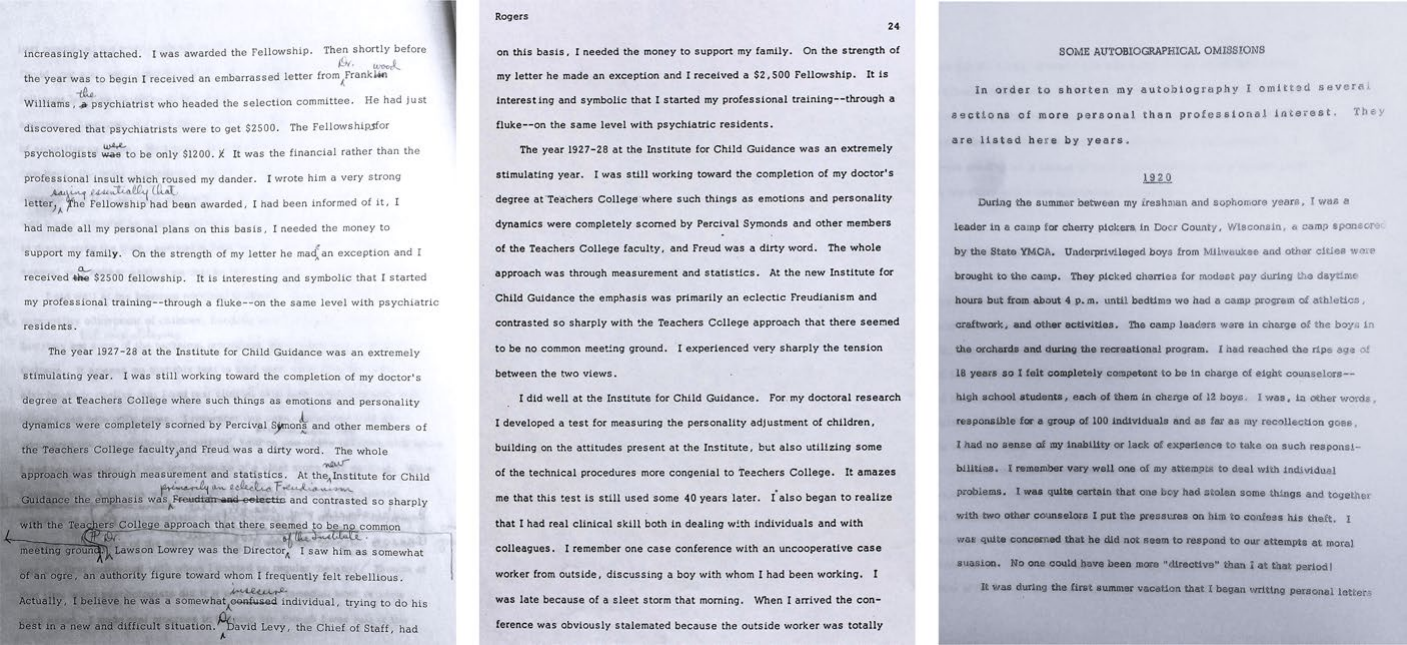
*Three versions of Carl Rogers’ autobiography*,* 1965-1978*. From left to right: untitled, heavily annotated draft written between 1965 and 1966 (later published as Rogers, 1967); corrected and edited “updated” 1971 version; and 1978 unpublished addenda documenting omissions. *Sources*: Carl R. Rogers Papers Collection, Box 161, Folder 5 (1965 draft) and Folder 4 (1978 addenda), Manuscript Division, Library of Congress, Washington, D.C.; Carl R. Rogers Collection, 1902–1990, Box 38, Folder 2 (1971 update), Department of Special Collections, Davidson Library, University of California, Santa Barbara. Images 1 and 3 in the public domain (Copyright given to the public). Image 2 reproduced with permission of the curator of the Carl Rogers Papers, UC Santa Barbara Library, Department of Special Research Collections.

Sustained contact with Rogers’ archival collections, notably the extensive holdings at the Library of Congress, clearly shows that his 1967 autobiography underwent multiple drafts and revisions. As shown in Fig. 1, the original version, written between 1965 and 1966 by request of Edwin Boring and Gardner Lindzey, is a combination of typescripts and handwritten notes, heavily marked by Rogers’ annotations. Decidedly less polished, this first manuscript is more direct, and occasionally confrontational than the published version: indeed, there Rogers describes some former superiors at the Institute for Child Guidance in the late 1920s as “ogre[s].”^9^ Four years later, Rogers ‘updated’ the account, now prefacing it with the revealing admission that “How I became the person I am is something of which I am not at all sure.”^10^

Autobiographies are artifactual also in a chronological sense. A self-confessed perfectionist, Rogers drafted yet another, unpublished manuscript around 1978. Titled ‘Some Autobiographical Omissions’, the manuscript delves on Rogers’ experiences during the 1920s. Importantly, here the author explicitly acknowledged having removed sections from the 1967 text “in order to shorten” it.^11^ Now, and without constraints, he could expand on his early life experiences, effectively reshaping what thus far was known about his young adulthood.

These overlapping versions form a constellation that must be analyzed collectively to assess reliability and authenticity and weave a plausible, comprehensive narrative. Selective reading of isolated documents, especially those neatly edited and filtered for publication, can only produce partial or distorted interpretations. The archival record instead demonstrates the contingency, incompleteness, and context-dependence of all autobiographical statements: no version is final, and no account is definitive. This underscores the historian's obligation to survey, compare, and assess the historical record in its manifold forms, rather than absolving him of careful analysis.

### Memories Can’t Wait: Recollection, Retrieval and Reliability

Assessing the integrity, verisimilitude, and consistency of sources is central to historical research, especially when dealing with ostensibly revealing yet methodologically fragile materials such as interviews, oral histories, and informal recollections (Araujo, 2014). These sources are, as Burnham (2003, p. 37) noted, always a function of both the subject’s integrity and the interviewer’s “scholarship.” Like autobiographies, oral histories are susceptible to partiality arising from bias, memory lapses, perseveration, and untrained interviewing (Zenderland, 2003). Much of the Rogerian historical canon relies on precisely such materials, making their critical examination essential.

Rogers’ autobiographical recollections have rarely been cross-referenced or interrogated as historical evidence. Instead, they are treated as if they accurately represent Rogers’ life “as seen through his own eyes” (Cain, 1987, p. 476). Even when inconsistencies have been noticed, they have been dismissed as minor or irrelevant (e.g., Guenther, 2022, p. 200, n. 38). Yet Rogers himself expressed doubts about the accuracy of memory, both in general and regarding his own. In the unpublished manuscript of his 1971 “updated” autobiography, he admitted that “the individual’s memory of his own dynamics is often decidedly inadequate.”^12^ In interviews with his biographer Howard Kirschenbaum that same year, he frequently remarked that his memory “was not accurate enough to really comment.”^13^ Any focused historian will find a plethora of factual errors in the statements Rogers began making in the 1980s about his life before the 1950 s (e.g., Rogers & Haigh, 1983, pp. 7, 9).

Beyond specific lapses, Rogers’ recollections display what historians of psychology identify as *perseverations*: the tendency for self-narratives to harden into formulaic scripts as interviews accumulate and the subject ages. As historian John Chynoweth Burnham observed, when historical figures feel “insecure in [their] memory,” they often fall back on earlier manuscripts or published accounts, repeating established stories to “refresh” memory and stabilize their identity (Burnham, 2003, pp. 20, 27). Over time, such repetition produces testimonial ossification: the subject becomes “trapped in [their] personal myth” (Burnham, 2003, p. 21). Historical accuracy is compromised as the subject becomes influenced by errors that have previously crept into those written accounts.

These dynamics are evident in *The Quiet Revolutionary* (Rogers & Russell, 2002), Rogers’ only published oral history and a key text in the Rogerian canon. Conducted between late 1985 and Rogers’ death in early 1987 by his former student and self-confessed “conver[t]” David Russell but only published fifteen years later, the book claims to be “the most extensive compilation to date” about Rogers’ life (Rogers & Russell, 2002, p. xxiv). Prefaced by an introduction written by biographer Brian Thorne (which only makes use of Rogers’ *On Becoming A Person* and Kirschenbaum’s biography as historical sources), the book is widely regarded as a paradigmatic source on his “wide-ranging readings, mentors, professional collaborators, experiences, and research results” (Dalton, 2022, pp. 239, 246; Martin, 2025, pp. 251–252), and frequently cited as a “vivid picture” of Rogers and his “intellectual clarity” (Kirschenbaum, 2007, p. 580).

Yet *The Quiet Revolutionary*’s evidentiary reliability is deeply compromised. Throughout, Rogers repeatedly acknowledges severe memory gaps, stressing that his memory was “not particularly good” (Rogers & Russell, 2002, p. 159), and exhibits trouble accurately recalling the first quarter of his life– the developmental period most crucial to the Rogers’ canon. In fact, Rogers confesses to being “amazed” by how little “visual memory, nor any memory at all” he retained of his high school years (p. 43). At several points Rogers clarified that he was not speaking “from memory, but from looking back at” the past (p. 113), even noting that later events “colored” earlier experiences so that key events “do[n]’t stand out in my memory” (p. 93).

In conversation with Russell, Rogers also admitted to refreshing his memory through documents and published accounts: “*If it weren’t for reading the journal that I wrote at that time* [late 1910 s] I would say I didn’t remember it at all. All I remember is from currently reading what I then wrote” (Rogers & Russell, 2002, p. 53, emphasis mine). More problematically, he also acknowledged consulting his own biographies, especially “Kirschenbaum’s book, [thanks to which] I’m reminded of things that I would otherwise have forgotten” (p. 51). This admission is historiographically consequential. It suggests that the oral history itself is doubly mediated: shaped both by memory and by the secondary literature *about himself* Rogers absorbed from the 1970s onwards. In a recursive twist, biographical accounts such as Kirschenbaum’s began informing Rogers’ own recollections, thus feeding back into later historiography as seemingly primary testimony.^14^ The subject’s memory, reshaped by its representations, re-enters the archive as evidence: a loop that exemplifies the self-referential logic of the Rogerian canon.

### Authority, Ascendancy, and Disciplinary Legitimation Through Appropriation

Historians of science have long emphasized that historiography is never neutral, particularly in the sense that narratives are shaped, tailored, and filtered by institutional and professional objectives and inclinations (Collyer, 2010; Schaffer, 1996). In particular, historians of psychotherapy and the clinical sciences have highlighted the stakes involved in constructing a suitable historical tradition that contributes to the writer’s or subject’s originality, respectability, efficiency, or other epistemic aims as conceived and valued within the field of contemporary practice (Rosner, 2018; Shamdasani, 2005). This means that both individuals *and* disciplines craft self-presentations that legitimize authority and preserve identity. What psychologists “know” about a historical figure is often “a function partially of the prevailing interests” (Burman, 2022, p. 24) of successive appropriations rather than a direct reflection of the historical record.

This ‘interested mediatedness’ is particularly visible in psychotherapy: a field fragmented into hundreds of schools where history often functions as disputed territory for justification and identity-building (Forner et al., 2025). More acutely, for client or person-centered therapy, whose practitioners have found themselves “in a weak position” with “few adherents” since the 1980 s (Raskin, 1990, p. 367), historical reconstruction operates as a strategy of cohesion and survival. Facing external criticism and internal division into multiple “forms” (Crisp, 2025, p. 2) or “person-centered tribes” (Moore, 2025, p. 9), the community’s detachment from mainstream academia has deepened its isolation and “lack of cross-fertilization” (Lietaer, 1990, pp. 19, 22). As Joseph (2025) concedes, the approach “has fallen into relative obscurity,” and is “kept alive by a relatively body of scholars” preserving Rogers’ ideas, albeit “only in a piecemeal fashion” (pp. 6, 35).

Within this context, historical reconstruction, especially the veneration of Rogers’ own words, serves to legitimize each narrator’s voice while restoring coherence and legitimacy to the field as a whole. This impulse echoes (and often directly references) Rogers’ own insistence that no external authority “can take precedence over my own direct experience” (Rogers, 1961, p. 24). Although originally a statement of personal subjectivity and *not* about theoretical validity or research procedure, the person-centered community has again literalized this remark into an epistemic principle, grounding its developmentalist historiography on first-person authority. As Dolliver observed, “Client-centered therapy can be seen as Rogers promoting for clients the stance that he had taken in his own life” (1995b, p. 137; see also O’Hara, 1995, p. 42). Demorest (2014, p. 149) extends this logic further, locating the origin of Rogers’ theory in his “childhood immersion in subjectivity.”

This exemplifies what Shamdasani (2005, pp. 6–7) calls the projection of a “biographer’s home-made psychology” into the historical record. Despite warnings that psycho-biographers should remain “eclectic and opportunistic rather than systematic” (Fancher, 1998, p. 99), the Rogers canon reproduces the very structure of its subject: subjectivity as authority, transparency as virtue, and lived experience as origin. The result is predictable. Despite gestures toward pluralism, Rogers’ statements continue to sanctify and unify a fragmented community, his figure serving as the “dominant” symbolic reference of the field (Lietaer, 1990, p. 22). Whether revered as founder or as a fallible theorist, he remains the axis of professional identity, his “secure faith in the authority of his own understanding [being what gives] his work *the power–the ring of truth*–that so impresses his readers, students, and colleagues” (O’Hara, 1995, p. 42). Authority, subjectivity, and truth thus coalesce, as person-centered therapists “feel that Rogers has had an immense influence on their personal/professional lives”.^15^

In this context, historiography becomes a disciplinary mirror. Rogers’ writings are continually invoked to sustain lineage and authorize divergent interpretations. And because his childhood is seen as the seedbed of person-centered thought, any source touching on it, autobiographical or biographical, is subject to appropriation. In fact, even authors critical of the field’s ‘tribal’ fragmentation (e.g., Crisp, 2025 and Moore, 2025) construct their own historical reconstructions from personal recollections and autobiographies, illustrating how history becomes a rhetorical weapon in intra-professional debate. Moore’s article, as its opening sentences make clear, is addressed to psychotherapy researchers: “The starting point for engaging with this topic was noticing how my heart would sink whenever I hear that a particular psychological therapy is ‘evidence-based’ or ‘researched’. I immediately question: in whose interest is this research being done?” (2025, p. 1). Crisp likewise critiques “the politics of evidence-based research” (2025, p. 2) as one of the starting points for his engagement with the life and work of Rogers.

In brief, sentimentality and critique seem to collide in the Rogers canon. The desire “to look into [Rogers’] eyes, to listen to the tales of his many voyages” and “see something of the magic in the depths of his personal waters” (Heppner et al., 1984, p. 15) exemplifies what Popperian historian of science John Wettersten (1975) warned as ‘justificationist historiography’: a Whiggish mode of narrative that vindicates the present by idealizing and rewriting the past, and disguises professional devotion disguised as theoretical explanation.

## Turning Inward to Turn Outward at Wisconsin in the 1950 s and 1960s: The Historical Situatedness of Rogers’ First-Person Accounts

As autobiographical narratives and sources are shaped by the discursive, professional, and intellectual contexts in which they emerge, historians must recognize that first-person documents serve purposes such as legitimation, boundary work, or critique. This calls for reconstructing their “richer and more multidimensional historical context[s]” (Zenderland, 2003, p. 78) to understand their meanings, referents and often unseen implications. Indeed, extracting significance from autobiographical statements requires acknowledging that historical data are part of a jigsaw puzzle and that historical actors simultaneously operated, as in a Rubik’s Cube, across overlapping communities (academic, clinical, religious, familial), institutions (universities, clinics, conferences, homes), and media (theories, practices, self-narratives).

By transitivity, autobiographies’ conditions of production shape both their accuracy and scope (Elms, 1997, p. 52). If, as Zenderland (2003, p. 99) poses, historical sources emerge from “a series of debates taking place simultaneously within different communities”, these communities have to be taken into account when reconstructing the past. In regards to the Rogers canon, Rogers’ autobiographical turn in the early 1960 s must therefore be read against the dramatic changes in his professional and institutional situation. Specifically, his move from the University of Wisconsin to California coincided with personal disillusionment and professional conflict which clearly colored his autobiographical tone.

Psychologist-historians often portray this transition as a simple one: “[w]hen he was over 60, [Rogers] resigned his most recent post at a Midwest university [the University of Wisconsin] to move to a private institute in California” (Demorest, 2014, p. 165; see also DeCarvalho, 1991, p. 23; Martin, 2025, p. 253; Moore, 2025, pp. 4–6; Palmer, 2025, pp. 530–531). Yet archival reconstruction reveals a more complex picture. Rogers’ disputes with psychologists at Wisconsin, tensions over his collaborator Charles Truax’s unethical research behavior, and the collapse of his group’s project on psychotherapy in schizophrenics (Garfield & Bergin, 1994, pp. 11–12) led to deep disaffection with academic psychology.^16^ The implosion of the Wisconsin project, long underexamined, marked what European Rogerians later identified as a “crucial moment” in the field’s history: the beginning of its exodus from universities and retreat from mainstream psychology (Lietaer, 1990, pp. 20–21).

Rogers’ correspondence from the period captures his frustration, especially over Truax. Writing in 1965 to Gardner Lindzey –who, significantly, had just commissioned Rogers his first official autobiography–, the Midwesterner described Truax as “a very ambitious person” seeking “power and authority”, noting he “did not know of any other person who has such positive qualities and such great potential along with behavior tendencies and personal characteristics which tend to destroy that potential.”^17^ Six years later Rogers confessed in private: “I lost more sleep and became more agonized over that [conflict] than any personnel problem I’ve ever been through.”^18^

This turbulence, rarely acknowledged in full in the Rogers scholarship, coincided with Rogers broadening his readership and audience, departing for the Western Behavioral Sciences Institute in 1963, and shifting toward social and collective themes. It also overlapped with Rogers’ warning his students against the interpersonal consequences of dogmatically adhering to his model, and (crucially) with the autobiographical texts that would define his posthumous image (Crisp, 2025; Mruk, 2023, p. 99; Raskin, 1990, pp. 368–369). These experiences almost certainly shaped the tone and content of his later self-portraits.

Rogers’ archival materials from the Sigmund Koch and G. Marian Kinget collections at the Archives of the History of American Psychology corroborate this contextual hypothesis. In 1954, while still at the University of Chicago, Rogers was invited by Koch to contribute to the APA’s *Project A*, a systematical analysis of psychology later published as *Psychology: A Study of a Science* (1959).^19^ Koch asked Rogers for an account of client-centered theory emphasizing “background factors and orienting attitudes” –intellectual and disciplinary, not biographical– such as “education, influence of other theorists, general currents of thought within the field or the culture at large [or] previous research history.”^20^ Rogers worked on this large chapter from 1954 to 1959, spanning his move from Chicago to Wisconsin and the beginning of his problems at the latter University.

**Fig. 2:**
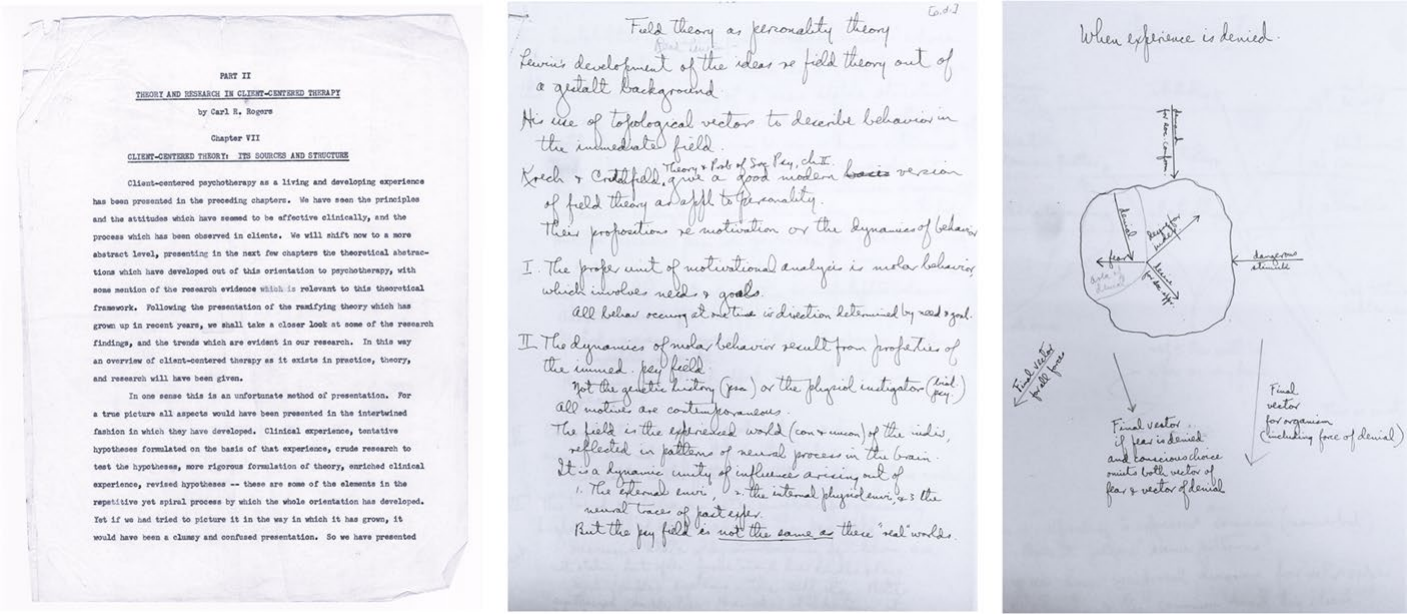
*Rogers’ working documents on the development and structure of client-centered theory*,* 1955-1962.* From left to right: Rogers’ original 1955–1956 draft of Chapter VII for Rogers and Kinget’s *Psychotherapie en menselijke verhoudingen* (Rogers, 1962); Rogers’ annotated working draft of his contribution to Sigmund Koch’s *Psychology: A Study of a Science* (Rogers, 1959); and Rogers’ drawings illustrating the graphical representation of experience denial prepared for Koch’s study. Sources: G. Marian Kinget Papers, Box M3308, Folder “Psychotherapy and Human Relationships,” Archives of the History of American Psychology, The Drs. Nicholas and Dorothy Cummings Center for the History of Psychology, Akron, Ohio (Chapter VII draft); Carl R. Rogers Papers Collection, Box 128, Folder 1 (annotations and drawings), Manuscript Division, Library of Congress, Washington, D.C. Images 2 and 3 in public domain. Image 1 reproduced with permission of The Drs. Nicholas and Dorothy Cummings Center for the History of Psychology, The University of Akron

Koch did not ask Rogers for autobiographical analysis. Yet in the final text, Rogers inserted the often-quoted statement that “No theory can be adequately understood without some knowledge of the cultural and personal soil from which it springs,” before describing his religious and familial background (Rogers, 1959, pp. 185–186). However, and as shown in Fig. 2, drafts from 1955 to 1956 reveal that these autobiographical sections appeared only later, precisely during the period of Rogers’ growing professional disaffection and academic conflict. Rogers’ 1955–1956 drafts were largely theoretical and epistemological, and Koch’s early feedback urged even greater formal rigor: “a discussion of the ‘life history’, so to say, *of a few of your pivotal constructs or hypotheses*”, not a discussion of the life history of the theorist himself.^21^ Rogers’ concurrent preparation of *On Becoming a Person* (1961) in the late 1950 s and his turn toward popular humanism, however, encouraged the blending of personal narrative and theory: the moment when self-reflection became both intellectual method and rhetorical strategy.^22^

Rogers’ collaboration with Belgian therapist G. Marian Kinget further illustrates this shift and confirms the very specific context in which Rogers’ most famous autobiographical account was produced. Five years after emigrating to the United States and joining Rogers at Chicago in 1950 (Mruk, 2023), Kinget collaborated with Rogers in the writing of “a book in French and Dutch which would present the client-centered point of view” to European audiences.^23^ The result was *Psychotherapie en menselijke verhoudingen: Theorie en praktijk van non-directieve therapie* [Psychotherapy and Human Relationships: Theory and Practice of Non-Directive Therapy], published in Dutch in 1960 and two years later in French.

Comparison of Rogers’ manuscripts from Kinget’s collection with the published editions shows a marked change in tone between 1956 and 1962. Rogers’ 1956 draft of one of the main chapters he authored, “Client-Centered Theory: Its Sources and Structure,” was a systematic, philosophy-of-science reconstruction of client-centered therapy. Here, as in with Koch’s project, “development” meant theoretical, not personal, evolution: the “repetitive yet spiral process by which the whole orientation has developed”.^24^ In fact, the early, manuscript version of this chapter traced back the system’s origins no further than Rogers’ early 1930 s Rochester years at the Society for the Prevention of Cruelty to Children, with no mention of his family or youth.^25^

Autobiographical content was a later addition, as evidenced by the manuscript’s inserts and references to the APA “request” (this is, Koch’s *Project A*) which were unrelated to the Kinget book.^26^ As a result, the Dutch and French editions of *Psychotherapie* (1960–1962) introduced explicit autobiography: Rogers (1962) now invoked “the widely held view that a thought [or theory] is better understood when one knows more about its author,” adding that some of his “opinions and convictions [were] deeply rooted in my experience” (p. 146, translation mine) before revisiting his Oak Park upbringing, his family’s ethos and his emotional background as a Midwesterner youth. Again, these insertions coincided with the Wisconsin crisis and parallel *On Becoming a Person*’s autobiographical tone: the moment when self-narrative became Rogers’ means of professional reorientation and private, personal restoration.

Together, these brief archival examples illustrate two key historiographical points. First, archival reconstruction transforms our understanding of ‘primary’ sources by anchoring them in their contexts. Second, and as a result, autobiographical statements must be read as historically situated literary productions shaped by circumstance, intention, and audience. As Zenderland (2003, p. 82) reminds us, the record really only is “a complex and very jumbled set of clues.” Only through integrative contextualization can a coherent picture emerge– one obscured when autobiographical fragments are taken as transparent, self-sufficient truths.

## Concluding Remarks: Enacting Historiography as Reflexive Practice

Throughout this paper I have argued that the historiography of Carl Rogers (the ‘Rogers canon’) embodies a recurrent and interconnected set of problems characteristic of insider, psycho-biographical historiography. At its core lies an epistemic tension between two impulses: a developmentalist, practitioner-driven reconstruction that treats first-person testimony as the most authentic source of historical truth, and a contextualist, archival approach that regards autobiography as an artifact requiring explanation. Left unchecked, the former turns biography into “a substitute, ersatz history” (Shamdasani, 2005, p. 7), whereas the latter restores the documentary and interpretive complexity the former obscures.

Three empirical patterns illustrate these problems. Rogerian historiography rests on a narrow evidentiary base: a small cluster of biographies and autobiographical writings has been repeatedly recycled with minimal archival verification. These narratives in turn display deep reverence for inherited sources: earlier biographies, Rogers’ own statements, and practitioner-driven accounts treated as definitive foundations of client-centered history. Finally, this dependence is further entangled with professional fragmentation and disciplinary interests: in practice, Rogers’ voice functions as a guarantor of theoretical legitimacy and collective identity, turning historiography into a tool of disciplinary legitimation and authorization. Beneath these factual tendencies, and notwithstanding their ultimate legitimacy as intellectual endeavors, lie deeper epistemological issues: a weak or rather naïve conception of evidence and a neglect of systematic archival grounding. Autobiographical materials, even when plain or candid, are artifactual, as in mediated by editing, omission, and retrospective reinterpretation, which in turn requires critical engagement and examination. Still, most accounts in the canon proceed without substantive archival engagement. A credible and nuanced historical reconstruction of Rogers’ life and work, even if aiming at collective cohesion and disciplinary legitimation, therefore demands multi-sited archival research, triangulation across collections, and sustained attention to alternative explanations and contexts.

The remedy to these issues is neither relativism nor the dismissal of autobiography as historical data. Rather, the current condition of the Rogers canon calls for a disciplined, reflexive historiography grounded in three commitments. First, *critical source practice* is imperative. Inherited narratives, especially practitioner-authored ones, should be approached with skepticism. Autobiographical texts should be treated as literary-historical productions whose versions, editorial interventions, and translations are documented and compared. Finally, when using interviews or oral histories, historians should reconstruct interviewer agendas, identify conditions of production, and assess possible self-inflicted “cribbing” from written documents (Burnham, 2003, p. 20).

Second, *contextual triangulation* is needed. First-person statements should be situated among contemporaneous records such as correspondence, institutional archives, drafts, and research files (Burman et al., 2025), and compared with scholarship outside the Rogerian canon whenever possible. Autobiographical turns must also be read alongside professional crises, institutional changes (such as Rogers’ Wisconsin conflicts), and publication imperatives: the ‘archival jigsaw’ method advocated throughout this article.

Third, *philosophical reflexivity and pluralism* should be assumed and encouraged. Historical data must not only be assembled but also interpreted, and its emerging image made meaningful and woven into coherent, truthful narratives (Zenderland, 2003). In order to avoid historiography “wandering around aimlessly” (Teo, 2013, p. 843), historians and their critics should make their own position, intended audience, and theoretical commitments explicit. (Coincidentally, a detailed, critical history of the Rogers canon and its many biographies *qua* documents is long overdue.) Attending to both research process and product thus enables the construction of plural, competing reconstructions rather than a single canonical life story to celebrate the subject and buttress the canon. Methodological heterodoxy (Mülberger, 2025) and a degree of ‘opportunism’ (Fancher, 1998) create the distance necessary to prevent unexamined narratives from hardening into disciplinary myth.

Crucially, none of this implies that Rogers’ autobiographical candor or the personal resonance of his work for contemporary practitioners is irrelevant. Autobiography matters, both as evidence and as a historical phenomenon. Furthermore, a critical approach to the canon does not mean dismissing or negating the rich, genre-defining scholarship produced by practitioners. The claim advanced here is narrower: autobiography, insider history, and psycho-biographical reconstruction are prone to share a set of epistemic perils. If autobiography is to serve as evidence in debates about the history of either clinical psychology, psychotherapy or personality theories, it must be interrogated, contextualized, and triangulated, and if it functions as a tool of legitimation, it should be historicized as such. Only through this disciplined, informed and self-aware mediation can historians draw warranted inferences about the relationship between life and theory without recursion or unwarranted teleology. Contemporary historiographic debates suggest such a cautious, contextually grounded approach is the only viable alternative to either uncritical, celebratory reverence or skeptical, tailspinning dismissal.

## Data Availability

All generated research data are available upon reasonable request to the author.

## Footnotes

1 I thank an anonymous reviewer for expressing this view in such a succinct and illustrative fashion. For a critical appraisal of overtly-critical takes on celebratory biographies, see the lengthy review by Söderqvist (2007).

2 Gondra’s recent article examining Rogers’ years at the University of Wisconsin in the late 1950 s and 1960 s (Gondra, 2025) was published after this article had been written, submitted and accepted for publication, which unfortunately precluded careful consideration of it and its incorporation into the body of historical scholarship analyzed here.

3 Robert Dolliver, “Carl Rogers’s Personality Theory and Psychotherapy as a Reflection of His Life Experience and Personality”, January 1993. Folder “Rogers, Carl”, Ludy T. Benjamin, Jr. Research Files, The Archives of the History of American Psychology, The Drs. Nicholas and Dorothy Cummings Center for the History of Psychology (hereafter ‘CUM’). Dolliver published his paradigmatic article on Rogers’ childhood and life in the *Journal of Humanistic Psychology* after his original 1993 version was rejected by the *American Psychologist*.

4 Dolliver, “Rogers’s personality”, 4.

5 Dolliver, “Rogers’s personality”, 4.

6 See also Dolliver, “Carl Rogers’ Personality Theory”, 6–8.

7 Dolliver, “Rogers’s personality”, 4.

8 Dolliver, “Rogers’s personality”, 4.

9 Carl R. Rogers, Untitled autobiography draft, 1965–1966, 31. Folder 5, Box 161, Carl R. Rogers papers, Manuscript Division of the Library of Congress, Washington, D.C., United States (hereafter ‘Rogers Papers’).

10 Carl R. Rogers, Autobiography (1971), 2. Folder 2, Box 38, Carl R. Rogers Collection, Department of Special Collections, Davidson Library, University of California, Santa Barbara, United States (hereafter ‘Rogers collection’).

11 Carl R. Rogers, “Some Autobiographical Omissions” (ca. 1978), Folder “Carl – Biographical Info”, Box 54, Rogers collection.

12 Carl R. Rogers, Autobiography (1971), 2.

13 Rogers, “Tape #02. Carl on College Years” [transcript], October 31, 1971, 30. Box 60, Rogers collection.

14 Russell himself expressed doubts about the genre after conducting the interviews, admitting that his contacts with Rogers “forced me to rethink the value of oral history […] [and] the whole notion of biography. Was it possible to reconstruct a life history, to uncover the wellspring, the inner voice?” (Rogers & Russell, 2003, p. xxiv).

15 Dolliver, “Rogers’s personality”, 5.

16 Garfield and Bergin’s chapter includes a brief summary of the Wisconsin fiasco. Their account is deemed authentic, or at least sufficiently trustworthy, since Bergin worked alongside Truax in Rogers’s project at Wisconsin during the early 1960s, so he had first-hand access to this controversy as it unfolded. For an overview of the conflict, see Gondra (2025).

17 Rogers to Gardner Lindzey, February 2, 1965, Folder 2, Box 6, Rogers papers.

18 Rogers to Howard Kirschenbaum, March 1971, 27. Box 60, Rogers collection.

19 Koch to Rosalind Dymond, July 22, 1954. Folder “General Correspondence, A-D”, Box M5023, Sigmund Koch papers, CUM.

20 [Sigmund Koch], “American Psychological Association. Study of the Status and Development of Psychology in U.S. Project A: Analysis of Psychological Science. Suggested Discussion of Topics for Contributors of Systematic Analyses”, 1, 7. Folder 1 Box 128, Rogers papers.

21 Koch to Rogers, July 8, 1955. Emphasis mine. Folder 1, Box 128, Rogers papers.

22 Paul [surname not given] to Rogers, June 26, 1955. Folder 1, Box 128, Rogers papers. Paul, a member of the private Pinel Foundation psychiatric institution, told Rogers that his sister-in-law had enjoyed Rogers’ recent paper, “something on ‘Becoming a Person’”. That Rogers had been planning the book for a while is also suggested by him in Heppner et al. (1984, p. 16).

23 Rogers to Koch, August 21, 1959, and June 27, 1956. Folder “Rogers, Carl”, Box M5030, CUM.

24 Rogers, “Client-Centered Theory: Its Sources and Structure”, 1. Folder “Psychotherapy and Human Relationships”, Box M3308, G. Marian Kinget papers, CUM.

25 Rogers, Client-Centered Theory: Its Sources and Structure, 2.

26 Rogers, Client-Centered Theory: Its Sources and Structure, 3–4.
